# Hotel Dieu

**Published:** 1830-04

**Authors:** 


### HOTEL DIEU.

### *Luxation of the Ulna backwards, with a Wound of the Elbow-joint: Hemorrhage: Cure.* By M. BOUDAULT.

THE radius and ulna are so securely united, that it was long doubted whether either of these bones could be displaced without the other. DUVERNEY first described dislocation of the radius. Sir ASTLEY COOPER is the only writer who mentions dislocations of the ulna alone; and he has given an engraving of this injury.* Cases of this kind must, therefore, be extremely rare.

François Alexis fell from a balcony upon the palm of his left hand. The arm supported the whole weight of the body, while the external part of the forearm, precisely at the part which corresponds to the coronoid process, and the superior extremity of the ulna, was seriously bruised and driven backwards by a large stone which accidentally lay where he fell. When he recovered from the faintness which was the first effect of the accident, severe hemorrhage took place from the wound. A surgeon attempted in vain to stop the bleeding, and he was taken to the hospital.

His face was pale and covered with sweat, and he had every appearance of having lost a considerable quantity of blood. The hand and forearm were in a state of flexion. On the external part of the elbow-joint was a wound, from eighteen to twenty lines in extent. Between the lips of the wound were observed shreds of ligaments and lacerated muscles. The trochlear surface of the humerus was seen smooth and polished. The olecranon was also distinctly seen and felt, elevating the skin and forming a considerable projection. Externally, the articulation presented no remarkable appearance. The radius was not displaced. In fact, while with one hand the arm was rotated, and the index finger of the other hand introduced into the wound, it was evident that the radius was in its natural situation, and in contact with the humerus. The patient was carefully examined by M. PAGÉS, as well as M. BOUDAULT, and there could be no doubt that the dislocation was of the ulna alone.

Reduction was easily effected by the same means that would be adopted for luxation of both the radius and ulna. The lacerated muscles were replaced as neatly as possible within the wound,

* Sir Astley Cooper, too, is the only author who has described, from personal experience, five species of luxations of the elbow, among which is that of the ulna backwards. Sir Astley mentions, 1, dislocation of the radius and ulna backwards; 2, of both these bones laterally; 3, of the ulna alone; 4, of the radius alone forwards; 5, of the radius backwards. No surgical library can be complete without Sir Astley's work on Dislocations. To country practitioners especially, who frequently cannot obtain a surgical opinion without considerable delay, it must prove invaluable for reference. —EDITOR.

which was allowed to unite by the first intention, in order to prevent the admission of air within the joint. The hemorrhage was arrested by light compresses, and simple dressing applied. The limb was then placed in a state of semiflexion upon a pillow. Low diet. Such were the means employed for this severe accident. The quantity of blood which had been lost rendered venesection unnecessary, in the opinion of the surgeons.

The next day the patient was calm. Pain not very severe, but, as the pulse had risen, he was bled to prevent inflammation, which was so much to be feared.

During the ensuing four days, no remarkable symptom occurred. The limb was neither tense nor swollen; tranquil sleep; the joint free from pain.—Soup was allowed.

The fifth day, when the dressings were removed, the lint was found covered with blood, but the wound presented a healthy appearance, and was rapidly healing. Fresh bandages and simple dressings were applied.

From this time the patient went on well, and in less than a month from his admission he left the hospital. A roller was applied round the arm, and the man was cautioned to be very careful of the limb. He promised to return if any new symptoms arose; but, as he did not make his appearance, it was presumed that the case terminated well.

This case, in which there was so much general disturbance from the accident; a wound penetrating the elbow-joint, excessive hemorrhage, and, above all, luxation of the ulna backwards, proves that a cure may sometimes be effected even in the severest accidents, in as short a time as when only a simple wound has been inflicted. The abundant hemorrhage, the immediate reduction of the dislocation, and the subsequent venesection, no doubt prevented those inflammatory symptoms which in similar cases so frequently run on to the destruction of the patient. The constitution of the patient, too, was very favorable for his recovery.*

* Revue Méd. Janv. 1830.

